# Undergraduate student perceptions of stress and mental health in engineering culture

**DOI:** 10.1186/s40594-023-00419-6

**Published:** 2023-04-24

**Authors:** Karin J. Jensen, Joseph F. Mirabelli, Andrea J. Kunze, Thomas E. Romanchek, Kelly J. Cross

**Affiliations:** 1 Department of Biomedical Engineering, University of Michigan, 1101 Beal Ave., Ann Arbor, MI 48109 USA; 2 Department of Educational Psychology, University of Illinois Urbana-Champaign, 1310 S. Sixth St., Champaign, IL 61820 USA; 3 Department of Counselor Education and Psychology, Delta State University, Highway 8 West, Cleveland, MS 38733 USA; 4 Department of Bioengineering, University of Illinois Urbana-Champaign, 1406 W. Green St., Urbana, IL 61801 USA; 5 Department of Biomedical Engineering, Georgia Institute of Technology, 313 Ferst Dr., Atlanta, GA 30332 USA

**Keywords:** Qualitative, Mental health, Stress, Interviews, Undergraduate, Student experience

## Abstract

**Background:**

Mental health for engineering undergraduates is an urgent topic for engineering educators. Narratives of engineering education requiring suffering may create or exacerbate problematic perceptions around stress and mental health in engineering. This study explored the roles of stress and mental health in engineering culture. We sought to explore: (1) how engineering students describe their experiences related to stress and mental health and (2) norms and expectations engineering students share about stress and mental health. Qualitative interview data were collected from 30 students who had previously responded to a college-wide survey.

**Results:**

Codes related to experiences with stress and mental health in engineering were organized in a bioecological systems model and analyzed for emergent themes depicting engineering culture. The study identified three themes related to stress and mental health in engineering culture: (1) engineering workload as a defining stressor, (2) specific barriers that prevent engineering students from seeking help for mental health concerns, and (3) reliance on peers to cope with stress and mental health distress.

**Conclusions:**

Our analysis provided insight into how engineering students perceive norms around stress and mental health in engineering and how this impacts help-seeking for mental health challenges. These findings have important implications for developing interventions and positive cultures that support student mental health.

## Introduction

Student mental health is an urgent and critical issue (Perkins et al., 2021; Xiao et al., 2017). Stress, a commonly discussed dimension of mental health, has been reported by studies of undergraduate and graduate students to occur at high levels during academic training (Ganesan et al., 2018; Karyotaki et al., 2020; Mackie & Bates, 2019). High stress levels can negatively impact academics (Pritchard & Wilson, 2003) and intention to remain in academic programs (Harris et al., 2016). While concerning in itself, high stress can further lead to additional mental health challenges and physical illnesses (Russell & Lightman, 2019). Experiences of high-stress cultures have been reported to be associated with anxiety and depression (Dohrenwend, 1998) for both graduate students (Bekkouche et al., 2022) and university students in general (Karyotaki et al., 2020).

National data indicate that diagnoses and severity of mental health disorders are rising for college students and that rates of depression and suicidal ideation are also increasing (Lipson et al., 2018). In parallel, college campuses have seen significant increases in mental health service utilization, rising from 19% of college students in 2007 to 34% in 2017 (Lipson et al., 2018). Despite increased utilization of services, many students are not receiving the individualized, specific, or targeted help they need. Studies have indicated many college students experience mental health distress; however, many do not receive proper treatment (Rosenthal & Wilson, 2008). The COVID-19 pandemic has further exacerbated students’ challenges around mental health care, with studies indicating a rise in college students’ perceived stress and mood disorders (Charles et al., 2021) and increased barriers to access to mental health care (Son et al., 2020; Wang et al., 2020). This rise in demand, barriers, and diagnoses highlights an urgency for developing more proactive supports for student mental health. Studies that specifically measured mental health for undergraduate engineering students have measured high rates of stress and mental health disorders (Danowitz & Beddoes, 2018; Jensen & Cross, 2021). Concerningly, studies have further shown that engineering students with mental health problems may be less likely to seek treatment (Lipson et al., 2016), underscoring the need to deepen our understanding of the engineering student experience related to mental health.

While research shows that overall stigma around mental health has decreased in recent years (Lipson et al., 2018), research has also shown that student help-seeking for mental health concerns varies by social identity, with some student groups feeling higher levels of stigma or experiencing other barriers to seeking help (Dai & Morgan, 2021; Han & Pong, 2015; Stebleton et al., 2014). In a 2017 study, Nobiling and Maykrantz identified sociocultural factors such as stigma, parental involvement and influence, or fear of lacking social support to be main reasons for students to not seek help (Nobiling & Maykrantz, 2017). This may be particularly true across cultures that overemphasize extreme resiliency, where social norms and expectations around enduring high levels of constant stress as a requirement for success may influence behavior around stress and mental health. The military history, glorification of poor self-care, and extreme working hours in engineering may contribute to students perceiving high stress and mental health challenges as essential parts of their engineering identity development.

Previous work has investigated levels of stress and other mental health challenges experienced by undergraduate engineering students, as well as experiences and beliefs related to help-seeking and mental health in engineering. Simultaneously, emphasis on rigor in engineering education has been critiqued as contributing to negative culture and exclusion (Riley, 2017). We propose that these themes of hardness and continuous struggle in engineering culture (Godfrey & Parker, 2010) may contribute to expectations of high stress and poor mental health as necessary in engineering programs, thereby impacting student behaviors related to mental health, including help-seeking. The present study expands on previous work to investigate beliefs and norms that engineering undergraduate students perceive related to stress and mental health through the lens of engineering culture. We posit that developing an increased understanding of the intersection of mental health, including stressful experiences, and engineering culture supports the development of proactive interventions to support engineering students.

## Literature review

### Engineering student stress and mental health

Our previous work indicated that many students perceived high stress and poor mental health to be normal and expected in engineering, which we have described as an engineering stress culture (ESC; Jensen & Cross, 2019). Students distinguished engineering programs as distinct from other academic disciplines in their beliefs that engineering students experienced higher stress compared to other academic disciplines (Jensen & Cross, 2019). Beddoes and Danowitz (2022) described high-stress cultures as leading to the “normalization and trivialization of mental health challenges” in which the widespread prevalence of stress results in the perception of mental health issues as “unimportant” (p. 4). Collectively, these findings suggest an ESC may contribute to normalizing poor mental health and experiences of high stress.

Previous research with undergraduate engineering students has quantitatively measured mental health dimensions (e.g., stress, anxiety, and depression) along with engineering identity and perceptions of inclusion in undergraduate engineering programs (Chase et al., 2022; Jensen & Cross, 2021), differences in prevalence of mental health conditions between engineering disciplines (Danowitz & Beddoes, 2018), help-seeking behaviors (Sanchez-Pena & Otis, 2021; Wilson et al., 2022), the influence of the COVID-19 pandemic on stress and coping (Beddoes & Danowitz, 2021; Danowitz & Beddoes, 2020), and stigma associated with mental health help-seeking (Sanchez-Pena et al., 2021). Limited research has explored undergraduate engineering students’ experiences (Beddoes & Danowitz, 2022) and shared cultural interpretations and understandings about stress and mental health (Mirabelli et al., 2020). While these studies are important in understanding undergraduate engineering student experiences, particularly regarding the use of mental health supports for students, less is known about the norms and expectations engineering students perceive for stress and mental health in engineering culture and the ways by which this culture is communicated and perpetuated.

### Culture in engineering

An organizational culture is composed of the shared norms and implicit expectations that define membership in a group, where aspects of the members of the group, such as actions and beliefs, are influenced by the culture (Schein, 2010). Individuals are acculturated to a group through their interactions with others and develop an understanding of these visible and invisible aspects of the group’s culture. Culture also impacts those looking in from the outside, shaping outsiders’ perception of those in the group. Collectively, culture defines normative behavior, which creates a positive feedback loop in further defining what is normal, acceptable, and expected. As such, culture can be both powerful in its pervasiveness and unseen in its ubiquity.

As a discipline, engineering is similarly defined by shared beliefs and norms about studying engineering and engineering as a profession (Godfrey & Parker, 2010). Engineering culture comprises the shared knowledge, values, and attitudes constructed by engineers (Godfrey, 2014). Engineering culture influences participation in the discipline, how the public perceives engineers, and how engineering is taught (Carberry & Baker, 2018). Recognized as a critical factor in the practice and advancement of engineering, culture is a focus of the National Academy of Engineering’s 2021–2026 Strategic Plan (National Academy of Engineering, 2021). Given the importance of culture, cultural studies of engineering aim to describe shared norms, beliefs, and values that are deeply rooted, yet often invisible to participants, that influence how people interpret their experiences and behave (Godfrey, 2014). Studies of engineering culture support understanding of shared beliefs and interpretations that guide behavior and are, therefore, requisite for developing new ways of thinking to create foundations for significant and sustained change.

Engineering culture has been critiqued as problematic in creating exclusionary environments and perpetuating the underrepresentation of women and students of color (Bix, 2014; Slaton, 2010). For example, engineering culture has been described as masculine, competitive, and White (Male et al., 2018; Pawley, 2019; Secules, 2017, 2019). This masculine culture and expectation of engineers as men creates challenges for women to be accepted and to be seen as “real engineers,” contributing to the “in/visibility paradox” described by Tonso where women are “highly visible as women yet invisible as engineers” (Faulkner, 2009, p. 169) and feel like outsiders (Tonso, 1999). Within engineering, subdisciplinary cultures have also been described, with some disciplines described as even less welcoming to women (Godfrey, 2007). Overarching engineering culture has been referred to as “meritocratic, exclusive, and elitist” (Secules et al., 2018, p. 80). These expectations of norms about who can be an engineer contribute to feelings about being an outsider for those who do not identify with these cultural norms (Foor et al., 2007). Feelings about being an outsider to engineering also contribute to challenges of retaining students in engineering. For example, in a 2012 study, Marra et al. identified feeling a lack of belonging to be a main factor contributing to students’ decisions to leave engineering majors (Marra et al., 2012).

In addition to defining who belongs in engineering, engineering culture further defines not only what success looks like but how one becomes successful as an engineer. In a narrative analysis of engineering culture, Sochacka et al. (2021) identified a dominant story of “making it through” engineering education programs, describing engineers as “heroes” who endure a challenging education and are rewarded with well-respected and high-paying careers (p. 68). This emphasis on endurance and determination through a grueling curriculum in what students perceive as a meritocratic environment (Rohde et al., 2020) emphasizes the notion of success through unrelenting hard work, supporting ideals of “hardness” in engineering (Godfrey & Parker, 2010, p. 12). Such narratives contribute to culturally constructed labels of students as “not cut out for engineering” and promote exclusion (Secules et al., 2018, p. 56). Defining success as unrelenting perseverance despite arduous circumstances further contributes to problematic assumptions about required working habits of engineering students.

In addition to expectations of inherent characteristics of engineers and their working requirements, engineering culture also is defined by what engineers value. Cech (2014) described a “culture of disengagement” with three ideological pillars that inform the way engineers approach, discuss, execute, and evaluate their work, resulting in nontechnical concerns being viewed as distinct and separate from technical work. These three pillars include (1) depoliticization, (2) technical/social dualism, and (3) meritocratic ideology and, according to Cech, decrease students’ commitment to social welfare over the course of their engineering education, resulting in students devaluing “social” competencies over “technical” skills in engineering.

Expectations and assumptions about inherent abilities and characteristics of engineers, what success in engineering requires, and what engineers value create the foundation of engineering culture. An exclusionary environment with overemphasis on rigor and hardness, where social competencies are devalued, may create problematic assumptions and behaviors related to stress and mental health more broadly. In this study we explore how undergraduate engineers understand stress and mental health in engineering culture and how these shared assumptions and expectations may influence behavior related to mental health.

### Purpose

This study explores the roles of stress and mental health in undergraduate engineering student culture. This qualitative study includes students’ depictions of their experiences with stress and mental health; in the design and analysis of this study, we emphasized student experiences of stress. As described above, stress is a component of mental health and experiences of high stress have been reported to be related to further mental health challenges. Our previous work has shown that large numbers of engineering students face mental health challenges and that students may associate high stress and poor mental health with engineering, viewing poor mental health as normal or even necessary for engineering students (Jensen & Cross, 2019). The purpose of this study is to identify characteristics of the cultural norms and perceptions around stress and mental health for undergraduate engineering students. Studies of culture seek to describe shared understandings of beliefs and norms that influence how people behave and make sense of their experiences (Godfrey & Parker, 2010). We propose that understanding cultural knowledge and beliefs held by students about stress and mental health will support the development of improved interventions and support structures for students. In this paper, we focus on negative attitudes and expectations of the culture within engineering programs related to stress and norms. We argue that to dismantle negative cultural narratives and replace them with asset-based narratives, it is essential to first acknowledge and understand these negative narratives. Following the well-known concept in psychology, we must acknowledge the problem to address it. The acknowledgment and deeper understanding of how a high-stress culture negatively impacts engineering students will support the development of resources and programs informed by cultural norms and perceptions to best support students for the purpose of improving how they seek help for mental health challenges.

## Theoretical framework

The guiding theoretical framework that informed our study was the framework of engineering culture developed by Godfrey and Parker (2010). Godfrey and Parker’s engineering culture framework was used in designing the study, including the interview protocol, as well as to interpret emergent themes.

### Dimensions of engineering culture

Organizational culture, while often seemingly invisible to those surrounded by it, influences our beliefs and behaviors (Schein, 2010). Developed from Schein’s cultural framework, Godfrey and Parker (2010) described culture specific to the engineering discipline, leveraging questionnaires, discussions, field notes, and documentary evidence to describe the understanding, values, and assumptions that were shared by engineering faculty, staff, and students. Godfrey and Parker’s ethnographic case study detailed six dimensions of engineering culture (Table 1). The framework has been leveraged in interpreting facets of engineering education, including disciplinary differences in engineering (Godfrey, 2014) and a recent study of engineering culture during the COVID-19 pandemic (Deters & Paretti, 2021). To investigate the roles of stress and mental health in engineering culture, we implemented an ethnographically informed approach (Creswell & Poth, 2016) and sought to describe beliefs, norms, and behaviors of engineering undergraduates related to stress and mental health.

**Table 1:** Six dimensions of engineering culture defined by Godfrey and Parker

Dimension	Definition
An engineering way of thinking	How engineers think, types of knowledge that are valued by engineers
An engineering way of doing	Perceived norms about how engineers work
Being an engineer	Beliefs about what characteristics are valued and necessary to be an engineer
Acceptance of difference	Homogeneity and diversity in values, norms, and beliefs
Relationships	Expectations around how engineers should interact with each other
Relationship to the environment	How engineering education is situated within the larger environment (e.g., university, engineering profession)

Adapted from Godfrey and Parker (2010)

## Analytical framework

We implemented Bronfenbrenner’s (1979, 2005) bioecological systems model as an analytical framework (Magana, 2022) to organize a coding scheme around individuals’ relationships to a culture. Bronfenbrenner’s bioecological systems model was used during our data analysis to create a structure within our common codebook, which organized aspects of engineering culture found within our interview data by systemic levels. In addition, we used the model to describe how stressors and supports are organized in students’ individual environments. As Schein (2010) described organizational cultures as influencing individuals’ values, behaviors, and beliefs in a structured way, we found Bronfenbrenner’s model to be a useful tool for depicting the structure of students’ surrounding cultures.

### Bioecological systems model

Bronfenbrenner’s (1979) bioecological systems model provides a structure for exploring mechanisms that influence an individual’s development. The theory has been widely applied to disciplines in social sciences, particularly in developmental psychology, child and family studies, and education (Tudge et al., 2009). Multiple recent studies have connected Bronfenbrenner’s model to the experiences of undergraduate students regarding topics related to this study such as culture and experience, motivation, and barriers to success (e.g., Garvey et al., 2017; Jones, 2018; Loh et al., 2021). Rather than developing new theories or ideas about student development, we applied the language of the model in this study as a lens through which to discuss undergraduate experiences related to stress and mental health that are specifically situated within engineering culture. We used Bronfenbrenner’s bioecological systems model to provide a taxonomy for understanding the relationship between engineering students and the surrounding culture and social environment. This model was applied to organize our results in a first round of analysis before contextualizing our results to the dimensions of engineering culture described by Godfrey and Parker (2010).

**Fig. 1:**
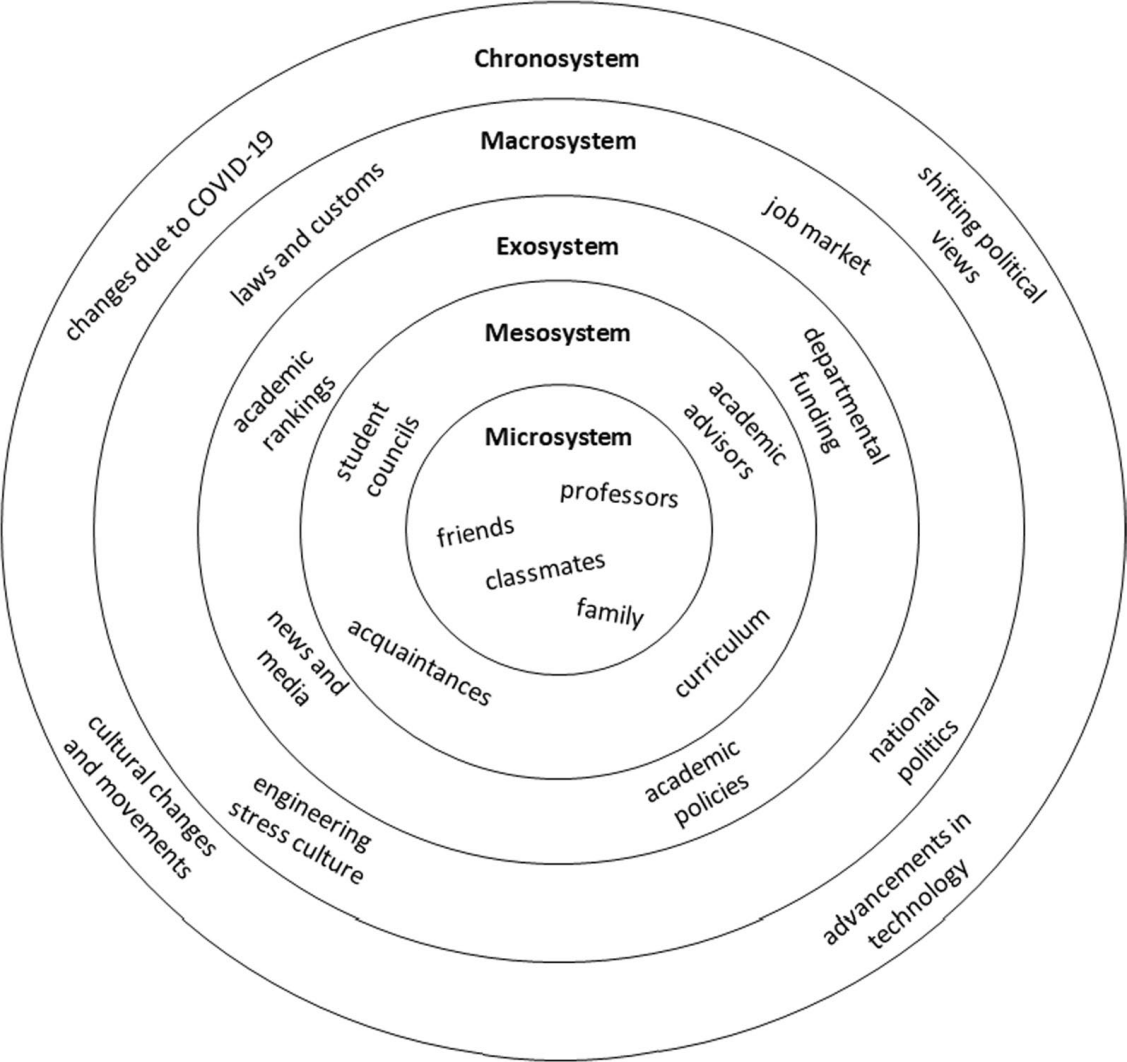
Example of Bronfenbrenner’s Bioecological systems model for college students

Bronfenbrenner’s (1979) bioecological systems model describes an individual’s growth by evaluating their interactions with their surroundings at five different environmental levels of decreasing proximity to the individual. The five levels of the model include (1) the microsystem, consisting of people and services directly and regularly affecting an individual; (2) the exosystem, involving an individual’s place within larger social structures or influences caused by those systems; (3) the mesosystem, located between the microsystem and exosystem and including elements of both, including both interactions between elements of the microsystem and interactions between the individual and a portion of their exosystem; (4) the macrosystem, including an individual’s place within broader present cultures; and (5) the chronosystem, the broadest level, which includes changes that occur over many years in broad areas such as national, international, and systemic cultures, defining ecosystems in more contextual ways (Bronfenbrenner, 1979; Tudge & Rosa, 2019). Figure 1 shows a schema of Bronfenbrenner’s bioecological systems model with general examples relevant to undergraduate engineering students. As this study occurred at a single point and did not analyze how engineering culture changes over time, minimal attention was given to the macrosystem in the development of a coding scheme and no attention was given to the chronosystem.

Bronfenbrenner’s theory has expanded as a result of widespread use to include more detailed processes; in this study, we applied a more recent extension of Bronfenbrenner’s theory including elements of the process–person–context–time (PPCT) model (Bronfenbrenner, 2005; Tudge & Rosa, 2019). The model extends Bronfenbrenner’s theory by including four components: (1) process, or specific actions constituting an individual’s interactions with a level of their bioecological system; (2) person, or specific biological aspects of a person such as race and gender; (3) context, or an individual’s presence in areas of their microsystem (e.g., students engage with engineering culture more at the university than at home); and (4) time, applying the role of time to each bioecological level (e.g., year in program on the microsystems level) as a further source of context (Bronfenbrenner, 2005; Tudge et al., 2009).

### Research questions

We explored the roles of stress and mental health in engineering culture by investigating the following research questions:

1. How do engineering students describe their experiences related to stress and mental health during their programs?
2. How do engineering students perceive norms and expectations around stress and mental health as part of engineering culture?

## Methods

In the present study we conducted semi-structured interviews with undergraduate engineering students to develop a shared understanding of the beliefs and norms engineering undergraduates have about stress and mental health. The data analyzed in this study are part of a larger, mixed methods project with the goal of better understanding of undergraduate engineering student mental health. We developed an interview protocol that drew on concepts of culture, with a focus on describing how students experience engineering culture as distinct from other disciplines, particularly regarding norms and expectations around stress and mental health. To include participants who varied in how strongly they identified with engineering culture, we intentionally stratified participants using a measure of engineering identity to ensure maximal variation of participants (Creswell & Poth, 2016). The one-on-one interviews allowed participants an opportunity to openly discuss topics that they may not have been comfortable sharing in a group setting. Both interviewers had familiarity with the setting as members of the college, but interviewers were matched with participants from different engineering departments. A total of 30 interviews were conducted with undergraduates at the focal institution. All study procedures were approved by the institution’s institutional review board before data collection began.

### Institutional context

Data for this study were collected from a large, public research university in the Midwestern United States. The institution offers 13 engineering degree programs across 12 departments and enrolls approximately 6000 undergraduate engineering students, with enrollment varying by major. The academic environment of the engineering college is often described by students as competitive, in both coursework and admission. Incoming students apply directly to engineering majors; the institution does not offer a common 1st-year engineering program.

### Recruitment and participants

We anticipated that the extent to which a student identifies with engineering may impact their experience of engineering culture, and that engineering departments and disciplines may also impact experience. We, therefore, leveraged a maximum variation sampling strategy (Creswell & Poth, 2016) and sought to intentionally stratify our sample by including participants with high and low engineering identity in addition to including participants from a range of engineering disciplines. Participants were recruited by email from the list of Fall 2017 respondents to a college-wide survey (Jensen & Cross, 2021). Participant recruitment and demographics were described previously (Mirabelli et al., 2020). Briefly, students who had responded to the survey were invited by email to participate in interviews based on measured high or low engineering identity measured by the Identification with Academics subscale translated to engineering (defined as upper or lower quartile for each department; Jones et al., 2010) and with the goal of representing as many departments in the college as possible (see section “Research Design”). In total, 150 students were invited to participate in the interviews and 30 students participated, which allowed us to reach saturation for the sample population (van Rijnsoever, 2017). Participants were compensated with a $30 gift card. Participants included 20 students who identified as women, nine students who identified as men, and one student who chose to not identify a gender identity. A total of eight engineering majors were represented. Participant information is summarized in Table 2.

**Table 2:** Table of participants

Alias	Engineering major	Gender identity	Engineering identity
Ralph	Materials	Man	High
Jasmine	Civil and Environmental	Woman	High
Rocco	Computer Science^a^	Man	High
Emily	Materials	Woman	High
Abby	Agricultural	Woman	High
Chelsea	Physics^a^	Woman	High
Amy	Materials	Woman	High
Caleb	Bio	Man	High
Lori	Civil and Environmental	Woman	High
Molly	Civil and Environmental	Woman	High
Josh	Computer Science^a^	Man	High
Anna	Aerospace	Woman	High
Katie	Bio	Woman	High
Nathan	Mechanical	Man	High
Ashley	Bio	Woman	High
Grace	Civil and Environmental	Woman	High
Allison	Physics^a^	Woman	High
Joe	Materials	Man	High
Becca	Computer Science^a^	Woman	Low
Ozul	Physics^a^	Man	Low
Talia	Bio	Woman	Low
Nas	Aerospace	Woman	Low
Georgina	Biological	Woman	Low
Victoria	Civil and Environmental	Woman	Low
Cecilia	Bio	Woman	Low
Chandler	Bio	Not specified	Low
Olivia	Bio	Woman	Low
Chris	Mechanical	Man	Low
Bradley	Bio	Man	Low
Richard	Mechanical	Woman	Low

^a^At the institution of study, the physics and computer science programs are housed within the College of Engineering, and thus we included students from these majors in our study

### Interview protocol

The development of the semi-structured interview protocol used in the study was described previously (Mirabelli et al., 2020). The interview protocol was field-tested with pilot interview participants external to the participant pool and reviewed in consultation with the project’s advisory board. The interview protocol was informed by the theoretical framework and designed to elicit norms and expectations in engineering, how engineering compared to other disciplines, and relationships (with peers and faculty). The semi-structured format permitted the interviewers to ask additional follow-up questions to address the research questions and explore emergent topics. The protocol included 22 structured questions across four main topics, including engineering identity and experience; perceptions of stress, anxiety, and depression; sources of stress in engineering; and coping and help-seeking behaviors (from both interpersonal and professional services). The interview questions can be found in the Appendix.

### Data collection

Participants were interviewed in person, in a private location on campus with one of the two interviewers. Interviewers reviewed and confirmed participant consent before beginning the interview. Interviews were audio recorded with permission of the participants. Participants were informed that the interview could be stopped at any point if they chose. Participants were asked to select a pseudonym or have one randomly assigned by the research team. At the conclusion of the interview, interviewers shared a paper copy of a campus resource sheet that provided information about the counseling center and other resources to support student mental health. Both interviewers completed field notes at the conclusion of each interview for process reliability (Walther et al., 2017) and dependability (Lincoln & Guba, 1985).

### Data analysis

All interviews were audio recorded and transcribed verbatim using a professional service, and a hybrid thematic analysis approach was utilized to examine the transcripts (Swain, 2018). In an initial round of open coding (Creswell & Poth, 2016), two research team members each individually reviewed ten transcripts and discussed similarities and differences between their preliminary codes and approaches to organizing the data. Ultimately, two superordinate groups of data emerged from the data discussions: (1) descriptions of stress as conceptualized by participants and (2) elements of engineering stress culture. The results from the former emphasize conceptualizations of stress as a process and are not presented here. The current study focused on the latter, and discussions across meetings between the two coders and the other research team members resulted in the development of an initial codebook based on these open coding themes, which were defined to align with Bronfenbrenner’s bioecological systems model. The initial codebook consisted of 11 themes containing a total of 28 codes, or clusters of meaning.

**Fig. 2:**
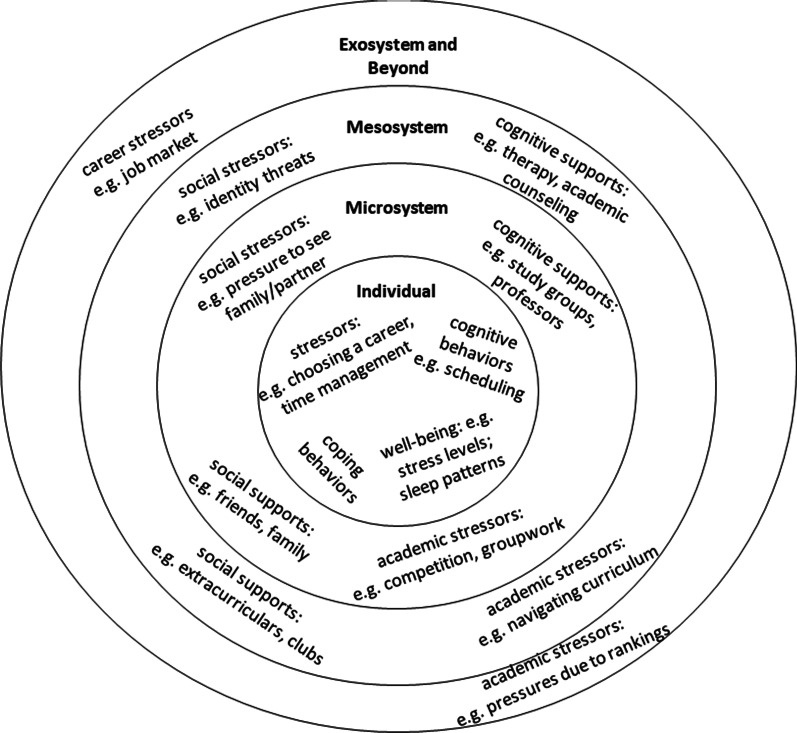
Application of Bronfenbrenner’s Bioecological systems model

This initial coding scheme, used in the first round of full coding, followed an organizational schema defined by Bronfenbrenner’s bioecological systems model. This schema was organized by levels in Bronfenbrenner’s model and included definitions of codes based on the model and its PPCT expansion (Bronfenbrenner, 2005) including aspects of individual students’ identities and personal contexts. The design of the coding scheme aimed to capture codes that emphasized engineering culture as it relates to stress and mental health, and was framed around the individual participant and the participants’ relationships with their own different ecological levels (e.g., mesosystem, self, macrosystem; Bronfenbrenner, 1979). Each systemic level in the coding system was divided into multiple aspects: sources of stress (code archetypes include self-initiated stressors, academic stressors, career-based stressors, social stressors), resources available and resources utilized for support (e.g., mental health resources, social or cognitive coping strategies), and climate (e.g., mental health norms, competition, effects of identity, perceptions of engineering versus other disciplines). The choice of Bronfenbrenner’s model came as a result of discussions between coders about how to frame the *sources of stress* appearing within the culture and *sources of coping or support*. These sources of stress were described as having consequences of various severity, from minor stress symptoms and rumination to serious mental health concerns such as debilitating anxiety. While participants did not often acknowledge or specify differences between stress and mental health, they described sources of coping and support both in terms of reducing the prevalence and effects of stressors and in terms of help-seeking (e.g., counseling and university accommodations, peer support) for mental health issues. The model, as a taxonomic framework, allowed the coders to discuss how the stress culture is organized and at what levels of interactions with the culture participants experienced stress or required additional support. Codes represented sources of stress, methods of coping with stress, and supports present in each ecological level. Figure 2 illustrates example codes using the multi-level model structure.

In the second stage of analysis and first implementation of full coding, we continued a priori and a posteriori coding; however, the same two researchers individually coded all 30 transcripts using the preliminary codebook. The researchers then compared each transcript for agreement and found differences in assigned codes and novel posteriori codes that emerged. In lieu of calculating inter-rater reliability scores, the coders employed a negotiated agreement approach. This approach allowed us to reductively develop a codebook for a large (30 interviews) qualitative data set with multiple researchers and account for discrepancies. The full corpus was then coded by a single knowledgeable coder (Campbell et al., 2013). In this approach, every intercoder disagreement in all coded segments across all 30 transcripts was discussed in multiple meetings between the two researchers until 100% agreement was met for all 30 transcripts. In this way, the two coders collaboratively formed a completely coded corpus of data organized by Bronfenbrenner’s bioecological systems model. During this process, the coders met frequently with the full research team for feedback on the research process and results.

In the final stage of analysis, a third member of the research team examined the fully coded transcripts to provide further refinement and finalization of the codebook by binning and organizing findings to align with Godfrey and Parker’s framework. This stage of analysis was primarily used in the description and organization of the discussion of our results. This researcher found that three dimensions of the Godfrey and Parker framework were particularly salient within the results. The researcher used the refined codebook to recode all 30 transcripts to finalize the list of captured text segments, adding their perspective as an undergraduate engineering student to the negotiated agreement process. Resulting from this process was a collection of captured text segments aligned with dimensions of the engineering culture framework; these segments were tagged with levels of Bronfenbrenner’s system and codes such as stressor or support type. Thus, this coding process “nested” the use of the analytical framework as a means of organizing the results to explore alignment with the overarching theoretical framework.

### Quality and trustworthiness

Throughout the research design and analysis, we followed quality procedures for qualitative research (Lazaraton, 2003; Walther et al., 2017). In addition, we reference established procedures for achieving trustworthiness in qualitative inquiry (Lincoln & Guba, 1985). The interview protocol was reviewed by an advisory board of three subject matter experts and was piloted with participants outside the sample to improve flow and clarity before use in data collection. Both interviewers completed field notes at the conclusion of each interview for process reliability (Walther et al., 2017) and dependability (Lincoln & Guba, 1985). Our interdisciplinary team held frequent meetings to discuss and revise codes and our analysis process for communicative validity (Kellam & Cirell, 2018). The coding systems and codes were frequently reviewed with the research team and discussed to achieve consensus. Finally, in translating our findings, we leveraged prior research, our experiences with engineering education, and the “informant role” of our engineering student experiences to triangulate congruence of our results as a form of establishing the credibility of our results (Lincoln & Guba, 1985).

### Researcher positionality

Drawing on Schein’s (2010) insight that “culture is not only all around us but within us as well” (p. 9), we recognize that our experiences and identities as a research team influence our interpretation of engineering culture. The research team consisted of five members, who at the time of data collection and analysis were two faculty principal investigators, two graduate students, and one undergraduate student. Four research team members (all but one graduate student) have experience as undergraduates and/or graduate students in research-intensive engineering programs and drew on their personal experiences to contextualize data. In addition, these four research team members all have experience in engineering as students, instructors, and/or researchers at the focal institution, which guided their interpretations of the influence of the institutional context and provided empathy, intuition, and understanding with participants’ experiences. The faculty team members contributed to the development of the interview protocol, leveraging their past and current experiences as engineering students and engineering instructors. The fifth research team member brings expertise as a doctoral student in educational psychology and has conducted extensive qualitative research studying student and academic climates in disciplines outside of engineering, which provided a contrast to help us understand unique aspects and experiences of engineering culture. The graduate student experienced with engineering is also a doctoral student in educational psychology. This coder was a former engineering student at the focal institution who experienced mental health distress during their undergraduate student experience; this coder’s experiences lent a sense of understanding in parsing student language related to mental health and stress. As coders, the two graduate student team members with educational psychology expertise chose Bronfenbrenner’s model as a taxonomic language related to cultures, because it was familiar to their understanding of culture and student experience, another way by which researcher experiences shaped the analysis of the data. The final coder was a current undergraduate engineering student at the focal institution. The coders leveraged their lived experiences as undergraduate engineering students toward their understanding of interview data. For example, the coders were able to understand vague language some participants used to describe aspects of the focal institution’s counseling center in terms of its reputation, its policies for scheduling appointments, and its location. During the analysis phases, the faculty team members met frequently with the coders to discuss the research process, offering feedback and advice and leveraging their knowledge of engineering students at the focal institution to ensure the communicative validity of our results. One of these faculty is an expert in qualitative research methods. In sum, our lived experiences as engineering students and instructors and our expertise in education and engineering methods are reflected in elements recognized as important to determining aspects of positionality, such as our ability to observe engineering students, our chosen methodologies, and our understanding of our participants and their experiences (Secules et al., 2021a).

## Results

Our analysis identified three main themes from student interviews in answering our research questions to describe engineering student experiences, norms, and expectations related to stress and mental health in engineering culture: (1) engineering workload as a defining stressor, (2) systemic barriers engineering students face in seeking help for mental health challenges, and (3) reliance on peers as a coping mechanism. We further categorize the second theme into three subcategories of systemic barriers, including physical, cultural, and informational. We describe experiences of these themes of engineering culture as processes or interactions that occur within various environmental levels per Bronfenbrenner’s bioecological systems model.

### Engineering workload as a defining stressor

Overwhelmingly, students in our sample cited the engineering workload as a significant (and expected) stressor that differentiated engineering from other disciplines; often workload was the most frequent and/or severe stressor experienced by participants. Students identified engineering-specific stressors at the individual (e.g., grades), microsystem (e.g., peer competition), and mesosystem (e.g., course availability) levels. Students cited that the stress from engineering workloads from these levels comes primarily from the high volume of assignments, challenging material, and time required to complete assignments. Students often referenced the inherent difficulty of engineering curricula. Students also contrasted norms within engineering to other undergraduate disciplines, thus demonstrating influence on their views from perceived cultures (e.g., exosystem and beyond). Jasmine shared:

In general, I think the material is a little bit harder to grasp. And it takes a little bit more effort to try to grasp it ... a lot of people have to go to office hours or have to make study groups or have to find some sort of external resource to get that done. (Jasmine)

Jasmine suggested that because the material is harder compared to other disciplines, it also takes more of a student’s time, often requiring extra hours dedicated to office hours, studying with other students, and seeking out external resources. Students referenced the intense workload of their programs coupled with extracurricular activities adding to packed schedules. Emily shared:

I think just in terms of sheer quantity wise, [we] tend to take on a lot of things. You know, you have a good sizable course load, but you're also doing this outside of school, and you’re also doing this. And I think that as things just pile up, the sheer quantity of things that people need to get done just definitely causes some, some stress there. (Emily)

Emily referred to the “good sizable course load” required in engineering compared to other nonengineering disciplines and the norm of students taking on additional roles and responsibilities outside of coursework (e.g., research). Grace explained that the rigorous course load leads to a shortage of time for other activities:

The course load of any engineering major is, really rigorous, and that requires a lot of work, both inside and outside of the classroom. And then there’s only so many hours in a day, and you wanna do all these things. (Grace)

Similar to Emily, Grace referred to extracurricular involvement adding to already packed schedules and the pressures of trying to be involved despite limited time. Nas agreed with this time pressure and summarized the experience as “having to do a lot of work and feeling like there’s not enough time to complete the amount of work that you’re given to succeed.”

Nas and other students in our study believed the engineering workload is more difficult because of the inherent complexity of the concepts and the amount of work. Students often discussed how engineering was “harder” and “more rigorous” compared to other majors. For example, Anna acknowledged that while she didn’t care for the stereotype of engineers as superior, she emphasized that STEM students work more compared to other majors:

And I hate, it comes with the old engineering attitude adage, that we think, you know, STEM people are more superior, and they have to do more work. But statistically speaking, engineering and STEM degrees are significantly harder, and you have a lot more workload, so I think that it’s definitely more stressful and harder on the students. (Anna)

Students emphasized that this heavy workload, combined with an expectation to be involved in many extracurricular activities, led to a constant time pressure that meant they had to work continuously. This resulted in students feeling that they didn’t have time for other activities. Emily explained that this “stereotype” of engineering students has some truth:

People joke, they’re like, “Oh, you only have time to go out and party every night because you're studying accounting. Of course you do, you don’t have that much to be like worried about you know,” but if you’re like an engineer, you’re like, I'm studying for calculus and physics and this all at the same time. Like I don’t have time to, you know, go to do fun, this fun thing like other people, I would say that they’re stereotypes but at the same time sometimes they ring true. (Emily)

Other students reiterated Emily’s stereotype of engineers as those without free time and with high stress from academic coursework. Becca described her peers:

I would say a lot of them are stressed. Because I have a lot of friends who always we will always complain about how much work we have and how much not enough free time that we have to do things. (Becca)

Becca referenced complaining to her peers about the workload and lack of free time, because engineering peers would understand due to their shared experience. Amy emphasized the different experiences of engineering students compared to other students on campus. When discussing these differences, Amy was asked if engineering students experience more stress compared to other disciplines. She shared:

Yeah. I think that’s an easy one to answer ... there’s also kind of a [stigma] that engineering students, or a cliché, I guess. Engineering students, you have to study a week in advance while business students can study the night before. (Amy)

Amy described as a cliché, the common statement that engineering students must dedicate more time and effort to their coursework compared to other students, and that this is equated with experiencing higher stress. Interestingly, Amy also referred to this as a stigma, invoking a more negative connotation of the shared understanding of engineering students experiencing higher stress from heavy coursework loads.

Overall, students overwhelmingly cited the engineering workload as a primary source of stress in their lives, affecting their time, stress levels, and peers. In terms of bioecological systems, courseload stressors originated from proximal sources (e.g., individuals, instructors, and peers), but less proximal, cultural influences reinforced the existence of these stressors through stereotypes and norms about engineering workloads. Students viewed their experiences with this heavy workload as distinct compared to other students on campus and emphasized that engineering peers understood this shared experience.

### Barriers engineering students face in seeking help for mental health challenges

Despite engineering students sharing that they experienced significant stress and mental health challenges, many also shared that they did not seek help other than from their peers. Our study identified themes of barriers students experience and perceive to seeking help for mental health challenges. These barriers included three categories: (1) physical (self and microsystem), (2) cultural (macrosystem and beyond), and (3) informational (mesosystem).

#### Physical barriers

Physical barriers to help-seeking resulted mostly from the students’ immediate environments (e.g., microsystems and selves): their schedules and available time, their locations on or off campus, and the moments at which their need for support was greatest. Consistent with student concerns about overloaded schedules, many students stated that they viewed help-seeking—for example, appointments at the campus counseling center—as another meeting or task that they needed to add to their schedules, including the challenge of last-minute scheduling. Ralph noted that the scheduling requirements created a barrier for him to go to the counseling center:

I know the counseling center requires you to call in the day of, to make an appointment. ... That can prevent you from doing that ... I know that’s part of ... also part of the reason that I haven’t gone to the counseling center and talked to them. (Ralph)

Students further commented that in addition to the extra time needed to meet with a counselor, there was the consideration of travel time to get to the counseling center, which is located on another part of campus. Georgina explained:

I think it’d be nice if people knew more about the resources at school. I know that there are, but I feel like most people don’t really reach out to get, get, to use those resources, because they might think, “Oh, it’s too far,” or it’s like, “Oh, I have to make time out, I have to loc—I have to allocate my time to address [these] mental issues.” Whether it’s stress or relationship things, so I think it’d be nice if it were more accessible. (Georgina)

Georgina referred to the need to “allocate” time to “address mental health issues,” emphasizing the time scarcity that students feel, and that while seeking help could alleviate distress, it would simultaneously cause additional distress due to a packed schedule. Talia described the “bother” to schedule appointments with the counseling center, adding that “it’s so far away.” The idea that resources are too far away geographically (due to noncentral locations) to make use of, on either a consistent or an infrequent basis, was commonly shared by students as a barrier that prevented them from seeking help.

Students further described barriers to accessing resources. Emily shared that she had heard of other students “not being able to get appointments even though they really need it.” Frequently cited was the limited appointment availability, lack of available appointments, or that appointments were limited to early morning hours. Ozul shared:

I personally don’t like the fact that you have to call in at 7:15 in the morning and book an appointment for the same day. And if it’s booked, they tell you to call again tomorrow. So, what is the point of me calling you guys today if you’re going to tell me ... I mean maybe my problem happened today and by tomorrow, if I don’t talk to someone, I might do something which I shouldn’t do. (Ozul)

Ozul expressed frustration about the limited appointments and the fact that when students need help the need is often at night and urgent. Talia agreed, sharing why she did not use the counseling center:

I think that the stresses, first off, a lot of them are stressed at night ’cause we’re doing homework, right at night. But I think that a lot of students, they feel like they need a counselor, and it’s usually an emergency situation where you’re like, oh I wish I had help, I don’t know. But most of us don’t take advantage and see the counselors regularly. And so, it’s hard to see the benefit and I guess for me I didn’t go in regularly enough that I saw the benefit either. (Talia)

Talia emphasized the fact that needing a counselor was often an “emergency situation” that could likely be at night when counselors are less available, and that most students did not seek out help proactively and instead may only reach out in a time of crisis, admitting that it was “hard to see the benefit” of seeking help outside of times of crisis. Talia noted that the times students often need support is at night, while they are studying or completing assignments. Also recognizing that the location of the campus counseling center and the time then required to use these resources was frequently cited by peers as a reason to not seek help, Lori shared:

So, I know the resource center, I personally don’t know where the resource center is located, but I think it would be nice like if during finals, during just stressful periods, like midterm season, if counselors are more available where people study. (Lori)

Lori noted that while she didn’t know where the counseling center was, she saw benefit in making counseling services available in the physical locations that students frequently occupy, as well as during times of higher stress (e.g., midterms). In addition to the perceived physical barriers to seeking help, some students attributed these barriers to insufficient funding for counseling services on campus. For example, when asked what he thought was needed to support engineering students experiencing high levels of stress, Rocco shared:

[F]or example the counseling center, I feel like there could be more funding for that. I think right now you have to make, you set an appointment at like, 8 a.m. Otherwise, you’re not gonna be able to get in. (Rocco)

Rocco, like other students in our study, indicated the challenge of appointments being limited and hard to schedule, and further indicated that this could be alleviated by the campus administrators dedicating more funding to mental health services.

#### Cultural barriers

While admitting undergraduate engineers experience significant stress and related mental health challenges, students cited high stress and even poor mental health as being normal and necessary for engineering students and reinforced by the norms and beliefs of the culture in engineering programs, describing the macrosystem and beyond. Emily explained high stress as being “part of the package” for engineering students:

Honestly, I think that high levels of stress is just part of the package when you’re an engineering student because the things you’re learning, you know, are hard, and you’re learning a lot of things at once. So, I think sometimes it’s just part of the package. There’s almost an attitude of if you’re an engineering student, and you’re not stressed, and it’s that’s really rare. That’s unusual. (Emily)

Molly explained that “people feel, ‘Oh, well, I'm supposed to feel like this. This is part of the discipline, part of the major.’” When questioned about what aspects of being an engineering student are stressful, Chris elaborated:

It’s honestly most of the culture. It’s when everyone else is saying this is what you should be working on, you should put this much effort into it, and you should be stressed, this is a very difficult position to be in. (Chris)

Chris’s comment that the expectations of work, effort, and stress for students create “a very difficult position to be in” emphasizes the pressures students experience to fit in and be seen as successful, and that students feel that if they are not stressed it is “unusual” or “rare,” as Emily described.

Despite this assumed norm of high stress and poor mental health, students described others as feeling “ashamed” to need help for mental health challenges. Cecilia shared, “I know a lot of people still don’t think it’s okay to just go talk... or to go to the counseling center. That’s a giant step though.... That’s admitting something you don’t want to admit.”

Cecilia described her peers as thinking it’s not “okay” to seek help for mental health challenges, reiterating the notion that many students shared of the expectation of engineering students to be resilient through mental health challenges, not wanting to “admit” that they need help, and that doing so would be a “giant step.” Amy described the façade that many engineering students maintain despite experiencing high levels of stress:

I think in general, a lot of students experienced stress but not a lot will talk about it. Just because there’s a sort of [stigma] that is around engineering students that they always need to be, they always need to push to be better. So a lot of people on the forefront act they’re doing really well in school, or act they all have all the time in the world. When in reality, everyone is kind of on that same playing field where everyone kind of needs help, or needs time off from school, or just kind of feel overwhelmed by school sometimes. But not a lot of people talk about it. (Amy)

Amy described the perceptions of what engineering students should be, beliefs that students are always high achieving and not in need of self-care or support, despite the fact that all people need both. Richard described this stigma of needing help, sharing:

I think ... a lot of times I encounter ... I have an interaction with students about this, they don’t necessarily take it that seriously. Or if they prioritize things above their mental health. They prioritize school and getting the absolute perfect grade above their mental health. And oftentimes I feel like ... if I ever suggested [to] them, go to the counseling center, just go see someone, I feel ... most people I ever talked to about that are pretty resistant to the idea ’cause ... I feel there’s just a stigma around it. (Richard)

Richard emphasized that his peers often prioritized academic achievement over mental health. Interestingly, in our study many students believed mental health resources would be or are effective for their peers but would not be or are not for themselves.

Students also described engineers as being “pretty bad” at dealing with “personal stressors.” Chelsea shared that engineers may bring stress on themselves, saying:

I think a lot of the stress, sort of, comes from ourselves. So, I think letting people who are highly stressed realize that, you know, it doesn’t have to be this way or there are ways to adjust, say either your perspective or even lifestyle to change that. So that you don’t experience that level of stress. (Chelsea)

Chelsea noted that students need to know that despite consistent cultural messaging that engineering students should experience significant stress, that “it doesn’t have to be this way.” Chelsea further shared that students need to be aware that not only is lower stress a possibility for students, but that there are some ways they can reduce stress in their own lives, including by altering their perspective to move away from the assumption of high stress and poor mental health as a necessity. In summary, participants in our study described shared expectations and norms of high stress and poor mental health in engineering, which discouraged student help-seeking for mental health concerns.

#### Informational barriers

In addition to the physical and cultural barriers to help-seeking, students described a lack of information about mental health resources on campus at the mesosystem level. Allison shared that while students are told that resources exist, they often don’t know the details about how to use them:

Here’s something that I don’t like. We’re told all of these things that you have, but you don’t really know where they are, how to get to them. I feel ... if I really had a problem, I’d call my parents or something. I wouldn’t know where to go or what to do, but I’m told we have a lot of resources. (Allison)

In addition to a lack of knowledge of where to access services, other students shared that they were confused about how services worked. Richard shared that he had gone to the counseling center, but wasn’t clear about procedures:

I went to ... the counseling center last semester when I was feeling very overwhelmed. ... I guess at first, I really had no idea what to expect and what exactly, the counseling center did. Was it supposed to be a long-term thing? Was it really just meet with them one time? (Richard)

Most students agreed that they had been presented with information about campus services during their education, but many did not remember details that would enable them to utilize the services. Ozul explained that students are presented with all the information about mental health resources during orientation, but students don’t remember specifics other than that resources exist: “’Cause during freshman orientation week, they explain all of [this] stuff to us but if something happens in sophomore or junior year, I’m not going to remember something that happened 2 years or 3 years back.”

Talia added that online trainings for students are not helpful and that students don’t “pay attention to them.” Overall, students were generally aware that resources were available, but admitted they were not sure how to use these resources, particularly when they had learned about them earlier in their education.

### Reliance on peers as a coping mechanism

While students in our study indicated they had minimal experience using campus mental health services, most shared that they relied on fellow engineering students within their microsystem to cope with high stress and mental health challenges. When asked if he had experience using campus mental health services, Ralph explained that he had not, and had heard from peers that the process was challenging. When asked about how he would manage a stressful situation instead, Ralph shared, “I'm pretty lucky to have a very good group of friends that I was able to go and talk to instead.” Nas explained how reaching out to peers was one approach that helped him cope:

Consulting with other people in my classes to kind of get a gauge on how they’re doing and how they’re feeling always helps me ’cause it makes me feel like I’m not alone. Um, but, yeah, that’s—that’s pretty much how I cope with stress. (Nas)

Nas referenced knowing how others are feeling and knowing that he was “not alone” when experiencing distress, again emphasizing the normalization of high stress. Caleb explained that the shared experience of high stress as an engineering student made peers the best option for coping: “I’d say the first line of defense, is definitely your peers. Because everyone else is likewise. If you’re stressed about something, you can find 20 people in a 50-foot radius that are also stressed out about the same thing.”

Molly explained that her approach to help-seeking was much the same: “Finding people that are in similar situations, I guess, so that you can talk about it to someone who can also relate and understands what it’s like.” Molly, like many students in our study, emphasized knowing “what it’s like” as an engineering student. Nas, Caleb, and Molly expressed that knowing that others were also experiencing distress was comforting, particularly due to the façade that “everything is fine” that Amy described in an earlier quote.

While many students expressed that going to other students was common to address their stress, some were hesitant to reach out to others. When asked if he would suggest that a peer seek help for a mental health challenge, Caleb answered:

Not usually, no. It’s very hard and I always feel if I can often tell when someone’s going through a rough time and I often have a hard time bringing it up and going like, hey I notice you struggling with this. ’Cause it’ll, it’ll all sometimes feel like you’re cornering somebody. It’ll feel like, oh I shouldn’t do that. That’s not very nice of me. (Caleb)

The fact that Caleb feels like he would be “cornering” a peer and not being “nice” suggests that some students may view unsolicited support for mental health challenges as unwelcome, despite the finding that most students describe themselves as reliant on peers for mental health support.

While students indicated a heavy reliance on their peers opposed to counselors, in contrast many students explained that instructors were generally not helpful for mental health concerns. Some students in our study often referred to the willingness of faculty and staff to help but emphasized that their help for mental health concerns was limited due to this lack of understanding. Nas explained: “The advisors are always, very... they try to be understanding but, at the end of the day, it always just feels like that’s their job and they don’t really fully understand.”

Nas referred to advisors wanting to help students and a perception that they do so because they feel it’s part of their “job,” but the lack of understanding from not being an engineering student limits their ability to provide support. In contrast to students relying on other peers who could relate to their experiences, students felt that faculty did not “fully understand” what they were going through. Faculty and staff populate the mesosystems and exosystems of students. Overall, they are less proximal to students compared with students’ friends or peers. Faculty relationships with students are generally less interactive and more situationally contextual to only academic concerns. Georgina explained these trends in terms of how she seeks support from friends or professors:

I personally go to my friends, roommates, if I have a—if I have any concerns or if I’m stressed about anything. If it is something more academic or career related, I go to my professors to ask their opinions and what can I do from there. (Georgina)

Georgina also emphasized the mentee–mentor relationship that students have with faculty, one that is limited to academics and careers. This involves viewing mental health as separate, despite students’ emphasis on high stress and poor mental health being “part of the package” that Emily described in a previous quote. Other students emphasized the variability in faculty members’ support of student mental health challenges. Becca explained: “It depends which faculty, too, because based on what I’ve heard some faculty members are not really helpful in mental health issues. And, they will add on to the stress most of the time.”

Becca further added that not only are some faculty not helpful for students with mental health issues, that faculty “will add on to the stress most of the time.” Other students shared that they wouldn’t feel as comfortable talking to faculty as they would talking to peers:

I don’t believe that I ever would [talk to faculty about mental health challenges] to be honest because I think that the faculty have their own, they’re all dealing with stuff and I feel more comfortable just talking to them about sort of coursework or anything regarded academically or research related, rather than discussing those sort of personal issues with them. (Chandler)

Interestingly, Chandler noted that “faculty have their own” mental health challenges, suggesting that the perception of poor mental health in engineering may extend beyond students to include engineering faculty.

## Discussion

If we understand the dynamics of culture, we will be less likely to be puzzled, irritated, and anxious when we encounter the unfamiliar and seemingly irrational behavior of people in organizations, and we will have a deeper understanding of not only why various groups of people or organizations can be so different but also why it is so hard to change them. (Schein, 2010, p. 9).

As described by Schein, studies of culture support our understanding of not only why groups are the way they are, but why they can be so persistently resistant to change. In engineering culture, the emphasis on rigor and toughness coupled with the devaluing of social competencies suggests that students may perceive mental health challenges differently, which may impact expectations, norms, and behaviors like help-seeking for mental health challenges. These norms and values of engineering culture are pervasive throughout bioecological systems levels. They are communicated through perceptions of culture at the macrosystem level and beyond and reinforced by the actions and habits of individuals and systems (e.g., peers, instructors, counseling services) at more proximal levels. Our study described student experiences and perceived norms related to stress and mental health in engineering programs. Schein (2010) described culture as the base of the “social order” and “rules” that members of a group follow (p. 3). In this study we sought to describe the “social order” and “rules” that undergraduate students follow and cocreate around stress and mental health. We discuss our findings within three dimensions of engineering culture: An Engineering Way of Doing, Being an Engineer, and Relationships (Godfrey & Parker, 2010) and compare and contrast our findings to related literature.

### An engineering way of doing

Undergraduates in our study emphasized that the intensity of the engineering workload was a defining stressor, often going to great length to distinguish this level of intensity and challenge from the experiences of non-engineering or non-STEM peers. This emphasis on workload as a defining stressor is consistent with previous work describing engineering as a “meritocracy of difficulty” (Stevens et al., 2007, p. 1) where learning engineering is described as “suffering and shared hardship” (Godfrey & Parker, 2010, p. 12). Students emphasized the never-ending lists of tasks and activities that left little to no “free” time in their schedules and often resulted in significant time management challenges. Godfrey and Parker (2010) described this approach in the cultural dimension An Engineering Way of Doing as a “just in time” culture where engineers often delayed working on tasks until right before they were due and, once started, continued working until the deadline. (p. 14). In addition to work often being last minute, descriptions of the heavy workload in engineering have included “horrific” and “living hell” (Godfrey & Parker, 2010, p. 12).

The intense workload in engineering programs placing limits on student time outside of coursework was commonly cited as a reason to not seek support. In a recent qualitative study with engineering students, Wilson et al. (2022) similarly described students deprioritizing mental health when they felt pressured by limited time.

Similar to beliefs shared by participants in our study about the necessity of long working hours, Blair-Loy and Cech (2022) described a “work devotion” cultural schema held by STEM faculty integral to merit. While this work focused on STEM faculty, it is likely that aspects of culture are cocreated by students, faculty, and staff in higher education and that faculty attitudes toward work may influence course design (and expected workloads) and interpersonal interactions around work habits and norms of stress.

### Being an engineer

In our study engineering students spoke proudly about being engineers, often emphasizing how engineering is “harder” and more “intellectually challenging” than other disciplines. In the Being an Engineer dimension, Godfrey and Parker (2010) described engineers as “tough,” where toughness characterized perseverance and determination instead of physical fortitude (p. 14). This emphasis on “tough” character may contribute to the cultural barriers to help-seeking observed in this study. In a recent qualitative study with 14 engineering students, Beddoes and Danowitz (2022) described this emphasis on toughness creating “an ethos of superiority that creates a culture of silence” where discussions of mental health challenges are suppressed to uphold the perceived ideals of engineers (p. 9). In the present study, students described the expectation of high stress and poor mental health as a reason to not seek help, since all of the students were experiencing the same high levels of stress. Previous work on help-seeking of engineering students identified themes of students feeling like they needed a “suck it up mentality unless they reach a breaking point” and that the need for an individual to seek help for mental health was shameful (Wright et al., 2021, p. 3). In our study, the ability to withstand the high stress that was defining of engineering was considered necessary and that failing to do so was a sign of not belonging in engineering. These beliefs about being an engineer, including what success as an engineer looks like, contributed to barriers students described in seeking help for a mental health challenge. Our study supported these findings that students may perceive increased barriers to mental health help-seeking due to cultural barriers based on ingrained norms about success as an engineer who is “tough” and has unwavering resiliency in the face of mental health challenges. While national studies have indicated that high rates of mental health challenges coupled with low help-seeking behavior is problematic across institutions, rates vary across campuses (Lipson et al., 2015), suggesting that campus and disciplinary cultures may contribute to this variability.

### Relationships

In the Relationships dimension of engineering culture, Godfrey and Parker (2010) emphasized the critical role of engineering peers in supporting academic success in time-consuming programs that left little time to interact with those outside of engineering. Our results supported this emphasis of reliance on peers in engineering programs. While Godfrey and Parker described this reliance on peers as necessary for academic success, we found that this dimension extended to support for mental health challenges, where students overwhelmingly shared that peers were their primary support. This aligns with Bronfenbrenner’s organization of bioecological systems, where interactions with and influences on individuals are most common within the microsystem, where peers are found. Barriers to help-seeking originating from proximal systems such as physical and informational barriers to counseling services contribute to this primary peer support status. Similar to how faculty co-construct an Engineering Way of Doing, evidence for this Relationship culture can be co-constructed by other engineering groups: in a qualitative study of coping strategies by Sallai et al. (2022) coursework loads were also described as a dominant stressor for engineering graduate students, and social supports were the most commonly used strategy to address that stressor.

Students shared that this reliance on peers was in part due to the shared understanding of the Engineering Way of Doing and Being an Engineer, that peers better understood the pressures and stress they were experiencing, which were unique to engineering students. While participants in the present study emphasized the reliance on peers to cope with stress and mental health challenges, other work has shown that peers may also be a source of support that promotes mental health help-seeking (Wilson et al., 2022). Collectively, these results suggest that for engineering students, peers are an important source of support for student mental health.

Students often referred to counselors, peers outside of engineering, or engineering faculty not being able to truly understand the unique experience of being an engineering student, and therefore, they viewed these individuals as less likely to be able to help when an engineering student was facing a mental health challenge. Godfrey and Parker (2010) noted that the relationships between faculty and students could be characterized as mentor–mentee instead of friendships, stating that students described faculty as usually “approachable and willing to help” (p. 17). This finding about faculty was in contrast to another recent study that described faculty lack of sympathy for and understanding about mental health challenges as contributing to mental health challenges and stigma (Beddoes & Danowitz, 2022).

### Limitations

We acknowledge that there are limitations to this study that suggest opportunities for future work. Our study design focused on a single institution, and we acknowledge that while many aspects of engineering culture are shared across institutions and programs, culture varies across institutions and programs. In addition, our interviews were conducted at a single timepoint during the students’ undergraduate programs and students’ views likely evolve over time. Furthermore, we did not collect information about students’ year in the program or age (beyond eligibility requirements), which could influence student experiences and perceptions about stress and mental health in engineering culture. In our analysis we used a negotiated agreement approach which allows for a level of inter-coder agreement but not inter-coder reliability (Campbell et al., 2013). We further recognize that students may lack clarity and use related but distinct mental health terms (such as “stress” and “anxiety”) interchangeably. Finally, our interview protocol included only some dimensions of mental health (e.g., stress, anxiety, and depression) and did not address other dimensions of mental health.

### Implications

Understanding barriers to students seeking mental health support is important in supporting students. The ideals of toughness and extreme resilience in engineering may discourage students from seeking help for mental health challenges. This may be exacerbated by the fact that some groups already feel increased stigma around mental health challenges (Masuda et al., 2012; Winograd & Rust, 2014). Together, these could in part explain why engineering students have been found to be less likely to seek help for mental health challenges compared to students in other disciplines (Lipson et al., 2016). As described by Emily, when students accept that high stress is “part of the package” of being an engineering student, they perpetuate the notion of a high-stress culture in engineering (Jensen & Cross, 2021). These results suggest that research studying undergraduate engineering student mental health and help-seeking should include measures of student identification with the expectation of high stress as a requirement in engineering.

The normalization of high stress in engineering, coupled with the heavy workloads that don’t leave time for mental health support and wellness practices, emphasizes the importance of normalizing wellness and self-care in engineering. Normalizing wellness and self-care in engineering will come from students seeing these practices valued not only by their peers, but also by their instructors (Jensen, 2021). Students in our study emphasized that they viewed their experience as unique compared to other disciplines, which suggests that mental health and wellness initiatives may be more impactful when delivered more proximally in engineering compared to at the campus level. Wellness activities integrated into engineering coursework (Miller & Jensen, 2020; Miller et al., 2022; Paul et al., 2020; Tait et al., 2022) or tailored for engineering students (Huerta, 2018; Huerta et al., 2021) may be important in demonstrating the importance of wellness to students as well as normalizing these discussions in engineering. Furthermore, integration of wellness into the engineering curriculum may counter the devaluing of social competencies described in engineering (Cech, 2014) and contribute to dismantling these aspects of engineering culture. This embedded wellness curriculum could include instruction on time management, which was a source of stress for many students in our study. Engineering faculty could consider structuring semester-long projects with additional check-ins to mitigate the “just in time” (Godfrey & Parker, 2010, p. 14) approach to engineering work. They could also be mindful about out-of-class time demands of projects that impact students’ availability for other pursuits. Furthermore, our results suggested that students need more regular reminders about campus services and their locations, with many students indicating that while they learned about the services when first coming to campus, they often did not remember the details when they needed support. To mitigate this, engineering faculty and staff could give more frequent reminders to students about campus mental health services, for example in syllabus statements and on course websites and university-affiliated social media. These reminders could simultaneously address the informational barriers described by students in this study as well as the cultural barriers by emphasizing the importance of mental health and wellness in engineering.

In addition to barriers perceived by students from the cultural ideals of engineering, students in our study also noted an inability to make time for mental health support due to their demanding schedules that they perceived as specific to engineering students compared to other disciplines. Students in our study routinely shared challenges (e.g., lack of appointment availability and time required to go to an appointment) that they had experienced when trying to seek help at the campus counseling center, or about experiences that they had heard from peers. These results emphasize not only the need for expanded funding for counseling services, as Rocco suggested, but also the need for increased availability and flexibility of appointments and appointment modalities (e.g., virtual). Students referred to often needing support at night or weekends when counseling services were not as readily available, as well as the distance of the counseling center from engineering buildings where students spent a majority of their time on campus. The emphasis on time as a barrier for engineering students suggests that approaches to reduce the time to access care may be particularly important to reaching engineering students. More flexible locations (e.g., engineering buildings) and times (e.g., nights and weekends) or embedded engineering counselors may not only meet these needs identified by students but may also increase the visibility and normalization of use of mental health and wellness services in engineering, which could contribute to decreasing cultural barriers that discourage help-seeking. Finally, this increased availability could prevent students from waiting until the situation is an “emergency situation,” as Talia described.

The most common coping mechanism shared by participants in our study was reliance on peers. The prevalence of this mechanism aligns well with Bronfenbrenner’s (1979, 2005) framework, as peers and friends are found within the microsystem or mesosystems, where degrees of influence and interaction are highest. In contrast, our participants expressed that they were unlikely to reach out to faculty and staff, who are generally in more distant mesosystem or exosystem levels. Godfrey and Parker (2010) described “academic survival and success would be very difficult for any student who was marginalized or a ‘loner’” (p. 16). The emphasis on the necessity of peer groups for success in engineering is likely compounded for students who are marginalized or feel a decreased sense of belonging. Reliance on peers may be emphasized due to the normalization of high stress, since students feel that the unique experience of being an engineering student can only be understood by their peers. The emphasis on peers being critical simultaneously for academic success and mental health support suggests that training and advocacy programs for engineering students may be part of an approach to supporting student mental health.

Indeed, most participants in our study stated they had not had an interaction with other students related to their peers’ mental health. This is likely connected to the façade that Amy and other students described of engineering students, who want to outwardly appear that they are unwaveringly resilient in the face of hardship. This façade is comparable to the engineering ideal of “tough, self-reliant, and capable, where toughness represents a personal strength of character rather than physical strength” described by Godfrey and Parker (2010, p. 14). In reenacting and reinforcing these ideals, students may unintentionally promote a culture where high stress and poor self-care is normalized, or even glorified in the competitive environment of engineering programs (Jensen & Cross, 2021). One example would be students bragging about the number of “all-nighters” pulled or hours worked. Some students in our study acknowledged the need for a culture change around mental health in engineering. Talia shared:

I think one of the things that really needs to be fixed on campus is the culture, the response culture. I think, even if you’re not going through something, all of you have a friend that’s going through something. But some people don’t know how to acknowledge or respond to that, and they make it worse, like a lot of times people make it worse. (Talia)

Providing students resources to refer to and advocate for their peers, and importantly, normalizing advocacy for other students, may be important in developing more supportive cultures in engineering.

Furthermore, efforts to research student stress and mental health, intervene in support of student help-seeking behaviors, and promote cultures of wellness should consider how the levels of students’ environments impact the amount and type of influence received. Peer group interventions, for example, may be promising as a more proximal form of support per Bronfenbrenner’s bioecological systems. While culture, including stress culture, is not proximal and, therefore, is difficult to influence, our work suggests that culturally constructed norms can reinforce stressors, that norms are communicated across levels of students’ environment (e.g., by conversations with engineering peers and instructors), and that stressors as well as supports or barriers to help-seeking are found throughout students’ environmental levels. Top-down interventions involving administrative or departmental policies or supports may be less immediately influential than bottom-up interventions that target students more directly.

### Future work

As noted in the Limitations section, there are limitations to this study that suggest opportunities for future research. Notably, the study represents a single timepoint and single institution. Perceptions of expectations and norms may change over time as students are acculturated in engineering programs, and culture may vary across institutions and programs. While this study focused on the experiences of engineering students, some of the findings may be generalizable to students in other academic disciplines. Additional research that compares and contrasts experiences of students from different academic disciplines will further our understanding of undergraduate student stress and mental health and inform tailored supports. Future work that includes additional institutions, timepoints, and students from more social identities will be important in increasing our understanding of the role of stress and mental health in engineering culture. Cultural barriers to help-seeking identified in this study, where students feel a barrier to seeking help due to expectations and definitions of success in engineering, could be studied using the framework of engineering students’ experiences of shame (Secules et al., 2021b). Students may feel shame about needing help for mental health challenges, since engineering students “should” be more resilient. Studies that include new barriers and norms influenced by the COVID-19 pandemic will also contribute to our understanding of the engineering student experience with mental health and contribute to the development of needed supports for students. Furthermore, the present study focused on the perspectives of undergraduates. Arguably, culture is co-created by all members of a group and future studies that include graduate student, faculty, and staff perspectives may enhance our understanding of how these expectations and norms develop and are reinforced by all members of an organization. In addition to understanding how we might dismantle the notions of a culture of high stress and poor self-care, future work that develops interventions to support a culture of wellness in engineering (Jensen, 2021) will support student thriving (Ge et al., 2021).

## Conclusion

In this paper we described a qualitative study of the roles of stress and mental health in engineering culture through the shared stories of 30 undergraduates. The study identified three main themes related to engineering students’ experiences and perceptions of stress and mental health: (1) engineering workload as a defining stressor, (2) barriers engineering students face for seeking help for mental health challenges, and (3) reliance on peers as a coping mechanism. These findings have important implications for the engineering education community to support a culture change where high stress and poor mental health are no longer viewed as necessary for success as an undergraduate engineer. Understanding students’ perceptions of stress and mental health in engineering and how these impact help-seeking behavior will inform proactive interventions to support student wellness. Furthermore, understanding how culture informs student decisions for help-seeking and facing mental health challenges and how barriers to help-seeking form in students’ environments can inform future work exploring student mental health, stress, and wellness initiatives. By changing the norms and expectations around stress and mental health for undergraduate engineering students, we can support students’ personal and professional success.

## Data Availability

As qualitative data were used, the data sets generated and analyzed in this study are not made publicly available to protect participants’ confidential data.

## Appendix

### Interview questions

Below is a transcript of interview questions as they appeared for the two interviewers. Before asking questions, the interviewers reviewed consent information and study procedures. After the interview questions, a short debrief including campus resources, remuneration information, and a future contact form were shared. The interviews were semi-structured, and the interviewers asked follow-up questions as they deemed appropriate. Prewritten probing questions were written to facilitate anticipated follow-up questioning.

1. What’s it like to be a student at your college? in your department?
  **Probe**: How do you see or describe yourself as a student?
2. How would you describe a “typical” engineering student in your college and/or department?
3. In your own words, how would you describe the characteristics of a professional engineer?
4. When you hear the word stress, what does it mean to you?
  **Probe**: How do you define stress?
5. Sometimes we use other words to indicate feelings of stress. What other words do you use to describe or talk about stress?
  **Probe**: Are there any other words that you use to refer to stress?
6. In your opinion, is stress different from anxiety or depression?
7. Can you tell me about a time or the last time when you felt stressed?
  **Probe**: Do you have examples of a stressful situation from your personal life?
8. What typically causes stress in your life?
  **Probe**: What are the things in your life that you think make you feel stressed?
  **Probe**: Are there things about where you live that you think make you feel stressed?
9. How would you describe the physical signs of your stress?
  **Probe:** Have you experienced any physical signs of stress such as hair loss, feeling tired, unable to sleep, weight gain, weight loss, persistent fatigue, back pain, headache pain, stomachache?
10. In your opinion, are there specific aspects of being an engineering student that are stressful?
  **Probe:** Do you think engineering students have more stress than students in other disciplines? What is similar and what is different? If so, why?
11. What characteristics of your discipline or department do you find stressful? Why?
  **Probe:** What are the challenges in being a student in your department?
12. What does it take to be a successful engineering student in your department?
  **Probe:** Are there habits or skills that are unique to engineering students to be successful that students in other disciplines may not need?
  **Probe:** Are there habits or behaviors that are rewarded or desired for students in your department?
  **Probe:** How did you learn about the habits or behaviors that are rewarded in your department?
13. Does your department strive to make everyone feel included? How?
  **Probe:** Is diversity and inclusion discussed in your department? How?
  **Probe:** What types of diversity or inclusion do you think your department is missing?
14. Do you *like and feel good about* being in your department?
  **Probe:** Does your department make you feel cared for and included? Why or why not?
  **Probe:** What makes you proud to be in your department?
15. In your opinion, what do you think it will take to be successful as a professional engineer? (e.g., habits, knowledge, skills, or abilities).
16. What aspects of a professional engineering job do you anticipate being stressful?
17. What resources and support are there on campus or in your department for students who are stressed?
  **Probe**: Have you utilized the mental health services provided by the university? Can you tell me about your experience with campus resources?
  **Probe**: Do you think that mental health services are helpful when you or peers feel stressed? Why or why not?
18. Can you describe things that you do personally to manage stress?
  **Probe**: Physical practices, Religious practices, or Well-being practices?
19. Are there coping strategies you find healthy or unhealthy?
20. Describe an interaction you have had with students regarding a mental health issue.
  <Examples for interviewer in case of confusion: loss of a family member or pet, arguments with roommates, etc.>
  **Probe:** Was the interaction positive? Why or why not?
  **Probe:** Was the student comfortable discussing a mental health issue?
21. Describe an interaction you have had with faculty regarding a mental health issue
  **Probe:** Was the interaction positive? Why or why not?
  **Probe:** Was the faculty comfortable discussing a mental health issue?
22. Based on your experiences, what would you say is needed to support engineering students experiencing high levels of stress?
  **Probe:** What advice would you give to incoming freshman engineering students about managing personal stress?
  **Probe:** What rule or policy would you create for promoting and supporting mental health for engineering students?
23. Is there anything else about stress in engineering that I didn’t cover that you wanted to discuss?
